# Intermediate syndrome following organophosphate poisoning: A case report in a low resource and poor socio‐economic setting

**DOI:** 10.1002/ccr3.6448

**Published:** 2022-10-08

**Authors:** Nimesh Lageju, Durga Neupane, Lokesh Shekher Jaiswal, Suresh Chapagain, Pratik Uprety, Swapnil Regmi, Silan Bhandari

**Affiliations:** ^1^ B. P. Koirala Institute of Health Sciences Dharan Nepal; ^2^ Department of Surgery (Division of CTVS) B.P. Koirala Institute of Health Sciences Dharan Nepal; ^3^ Chitwan Medical College Bharatpur Nepal

**Keywords:** atropine, intermediate syndrome, organophosphorous, poisoning, pralidoxime, mechanical ventilation

## Abstract

Intermediate syndrome with respiratory failure is a serious complication that can be fatal as in our case of a 24‐year‐old‐man who developed intermediate syndrome requiring intubation and respiratory support. Furthermore, the patient's socio‐economic situation significantly impacts the illness's progress and prognosis.

## Abstract 2

With this case report, we want to stress how a patient's socio‐economic background affects the course and outcome of a particular disease condition.

## 1 INTRODUCTION

According to World Health Organization (WHO), approximately 3 million pesticide poisoning occurs worldwide and more than 40,000 deaths per year.^1^ Among pesticide poisoning, organophosphate(OP) poisoning is one of the most common causes of poisoning in developing countries.^2^ Based on the onset of the signs and symptoms of OP poisoning, it is categorized into acute (minutes to 24 h), delayed (24 h–2 weeks), and late (beyond 2 weeks).^3^ The activity of muscarinic and nicotinic receptors causes acute onset symptoms. Muscarinic symptoms include salivation, lacrimation, urination, defecation, gastric cramps, emesis, bradycardia, hypotension, miosis, and bradycardia. Weakness, fasciculation, cramps, and paralysis are all nicotinic symptoms. The delayed onset nicotinic symptoms are intermediate syndrome.^4^ Intermediate syndrome (IMS) usually develops after 2–4 days of oral OP poisoning. It occurs in around 20% of OP poisoning cases. The paralysis of respiratory muscles and paralysis of the peripheral limbs are two of the most common symptoms of IMS. The treatment of IMS involves only mechanical ventilation for 7–15 days.^5^

## 2 CASE PRESENTATION

A 24‐year‐old‐male patient was brought to our hospital's emergency department with an alleged history of intake of organophosphate insecticide (Chlorpyrifos) in a suicidal manner. He presented with complaints of vomiting and a burning sensation over the epigastric region. He was partially conscious with a GCS of 9/15. He also exhibited miosis, hypersalivation, and bilateral crepitation on the chest. Gastric lavage was done immediately. The dose of atropine was begun at 3 mg (five ampoules) and was raised every 5 min until complete atropinization was attained. A total of 180 mg (300 ampoules) of atropine was required for atropinization and then the patient was kept under a maintenance dose of atropine. Injection pralidoxime was also administered. The patient was subsequently referred to a tertiary care center due to the necessity for intensive care. On the second day of intensive care unit (ICU) admission, the patient was unable to elevate his head and his saturation level had reached 80%. A diagnosis of the intermediate syndrome was made. Then, the patient was intubated and kept on a ventilator along with supportive care. Due to financial problems, the patient party could not afford the ICU expenses and was forced to leave the center against medical advice with an endotracheal tube in situ. The patient returned to the emergency department of our hospital. Because our hospital lacks ventilator equipment, the patient party had been providing bag and mask ventilation for nearly 48 hours, turn by turn. After raising sufficient funds, the patient was referred back to the tertiary care hospital for the need for ICU. Unfortunately, the patient died on the way to the hospital.

## 3 DISCUSSION

One of the most common poisonings seen in Emergencies in developing nations is OP poisoning, which necessitates close monitoring and prompt treatment. It manifests as a variety of muscarinic, nicotinic, and central nervous system symptoms. The most prevalent symptoms of OP poisoning are SLUDGE (salivation, lacrimation, urination, defecation, gastric cramps, and emesis). These muscarinic and nicotinic signs and symptoms are used to clinically diagnose OP toxicity.^6^, ^7^ The intermediate syndrome (IMS) is characterized as a muscular paralysis that occurred in patients 24–96 h after ingestion of poison. Muscle weakness primarily affects the muscles of the proximal limbs and those supplied by the cranial nerves. It is frequently associated with respiratory failure. Recent research suggests that IMS can arise before 24 h and after 96 h.^8^, ^9^, ^10^ Acetylcholinesterase aging, poor rephosphorylation and diminished synthesis of new enzymes have all been linked to delayed symptoms. If IMS presents with respiratory failure, it has a high fatality rate. Treatment of IMS is mainly supportive, early aggressive gastrointestinal decontamination, followed by adequate atropine and oxime therapy and timely initiation of ventilator support, should help to reduce the severity of IMS.^11^, ^12^

The most expensive days in the intensive care unit are the first 2 days, after which they stabilize at a lower level. For patients undergoing treatment in the intensive care unit, mechanical ventilation is linked to significantly higher daily costs throughout their stay. Interventions that minimize the length of stay in the critical care unit and the duration of mechanical ventilation could result in significant savings in total inpatient costs.^13^ However, the patient in our case was unable to afford to stay in an intensive care unit with mechanical ventilation and had to rely on the bag and mask ventilation until he lost his precious life on the way to the referral center.

This case report is significant from the standpoints of both the physician and the patient. The physician must diagnose IMS promptly and intervene or refer patients on time. And the patient's socio‐economic situation, as evident in our case, significantly impacts the illness's progress and prognosis.

## 4 CONCLUSION

The intermediate syndrome is a very prevalent complication of OP poisoning. If a patient develops respiratory failure, there is a substantial risk of death. Hence, a timely clinical diagnosis of the syndrome is very necessary. Supportive therapy is a cornerstone of IMS treatment. Mechanical ventilation should be started as soon as possible, along with appropriate atropine therapy. Aside from that, the government should safeguard those who are financially challenged, and only then will these deaths be avoided.

## AUTHOR CONTRIBUTIONS

NL, DN, LSJ, SC, PU, SR, and SB wrote the initial draft of the manuscript. NL and DN edited the draft and reshaped it into this manuscript. The final version of the manuscript was approved by all authors and agree to be responsible for all aspects of the work.

## FUNDING INFORMATION

The authors received no financial support for the research authorship and/or publication of this article.

## CONFLICT OF INTEREST

The authors declare no conflict of interest.

## ETHICAL APPROVAL

None.

## CONSENT

Written informed consent was obtained from the patient's party to publish this report in accordance with the journal's patient consent policy.

## Data Availability

All data about the case are available as a part of the article and no additional source data are required.
